# Evaluation of loco-regional recurrences using deformable image registration after isotoxic high dose stereotactic body radiotherapy in localised pancreatic cancer

**DOI:** 10.1016/j.ctro.2025.101081

**Published:** 2025-11-19

**Authors:** Martin Manderlier, Sara Poeta, Jean-Luc Engelholm, Akos Gulyban, Jean-Luc Van Laethem, Christelle Bouchart

**Affiliations:** a Department of Radiation Oncology, Hôpital Universitaire de Bruxelles (HUB), Institut Jules Bordet, Université Libre de Bruxelles (ULB), Rue Meylemeersch 90, 1070 Brussels, Belgium; b Department of Radiation Oncology, CHU de Charleroi, Boulevard Zoé Drion 1, 6000 Charleroi, Belgium; c HUB Institut Jules Bordet, Medical Physics Department, Brussels, Belgium; d Department of Radiology, Hopitaux Iris Sud, 1190 Brussels, Belgium; e Department of Gastroenterology, Hepatology and Digestive Oncology, HUB Bordet Erasme Hospital, Université Libre de Bruxelles, Route de Lennik 808, 1070 Brussels, Belgium

**Keywords:** Stereotactic body radiotherapy, Pancreatic cancer, Radiotherapy, Loco-regional recurrence, Deformable image registration

## Abstract

**Background and Purpose:**

Pancreatic ductal adenocarcinoma is a highly aggressive malignancy with poor survival outcomes. Neoadjuvant treatments, including radiotherapy, have been developed to improve resectability and survival rates. This study evaluates loco-regional recurrence (LRR) patterns after isotoxic high-dose stereotactic body radiotherapy (iHD-SBRT) for localized pancreatic cancer using deformable image registration (DIR).

**Materials and Methods:**

Patients with borderline/locally advanced pancreatic adenocarcinoma treated between January 2018 and January 2021 were included. Treatment involved modified FOLFIRINOX chemotherapy, iHD-SBRT, and surgery, if feasible. LRRs identified via CT/MRI during follow-up were mapped back onto initial radiotherapy planning using validated DIR workflows. Recurrences were classified as in-field (IF), marginal (M), or out-of-field (OF).

**Results:**

Among 41 patients, LRRs were identified in 17 (10 via CT, 7 via MRI). The majority of LRRs were classified as OF (53 %, n = 9), with 23.5 % (n = 4) each in IF and M categories. Marginal recurrences were located near major abdominal vessels, such as the superior mesenteric artery and coeliac artery. DIR quality metrics, including DICE and MDA, validated the accuracy of the mapping process.

**Conclusion:**

Image based evaluation of loco-regional recurrence is an important part of the assessment of patients treated with iHD-SBRT for localised pancreatic cancer. DIR-based analysis emphasized the importance of expanding the tumor-vessel interface (TVI) structure in radiotherapy planning to include the full circumference of adjacent vessels with a 5 mm margin. DIR-based analysis highlighted that several marginal recurrences occurred in proximity to perivascular regions, suggesting a potential benefit of expanding perivascular target coverage in future treatment planning.

## Highlights

• Deformable image registration was used to precisely map recurrence patterns.
• More than half of loco-regional recurrence were classified as out-of-field.
• The TVI structure include the circumference of abdominal vessels with 5 mm margin.
• Ongoing investigations are important to optimize neoadjuvant therapy protocols.

## Introduction

With an estimated 5-year overall survival (OS) of 12 % for all stages, pancreatic ductal adenocarcinoma (PDAC) is one of the most aggressive tumour and is responsible for nearly 44,000 deaths in Europe annually, making it the fourth leading cause of cancer-related mortality classification [1,2]. For a curative intent, a complete oncological surgery with microscopically complete (R0) resection is required [3]. However, only around 15 % of cases are classified as initially resectable. Other cases are classified as borderline resectable (BR, approximately 15 % of cases), locally advanced (LA, approximately 25 %), or metastatic. Additionally, a substantial proportion of resected patients have positive margin (R1) resection (depending of the definition of positive or close margin), with a significantly poorer prognosis compared to surgery with R0 resection [[4], [5], [6]]. For years, neoadjuvant treatments combining chemotherapy and/or radiotherapy have been explored with the aim of improving resectability.

Over the past 10 years, stereotactic body radiotherapy (SBRT) technique has been recently explored in several observational or phase II trials, allowing the delivery of ablative dose over a few sessions at the primary tumour site and easily fitting into a total neoadjuvant strategy (TNT) [7,8]. Following the negative results from the Alliance trial, efforts have been made to improve the SBRT technique notably by delivering higher biologically effective dose (BED), superior to 60–70 Gy, which was identified as a predictive factor of prolonged survival in several study [7,[9], [10], [11], [12], [13], [14]]. An SBRT based on an isotoxic dose prescription is a fitting technique to be able to deliver a high BED_10_ to the tumour without impairing the safety of the critical closest gastrointestinal organs at risk (OARs). We previously reported the promising oncological outcomes of localized pancreatic cancer patients treated with a TNT including FOLFIRINOX and isotoxic high-dose SBRT (iHD-SBRT), leading us to launch a randomized multicenter phase II study, the STEREOPAC trial, currently active for recruitment (NCT05083247) [7,15].

However, several previous studies involving SBRT for the treatment of localized PDAC have reported non-negligible rates of locoregional recurrence (LRR), mostly occurring outside the Planning Target Volume (PTV) [12,16]. This suggests that the smaller volume treated with SBRT may lead to an inadequate coverage of crucial regions at risk of microscopic disease compared to chemoradiotherapy (CRT) where an elective nodal irradiation is usually performed.

The aim of this study was to perform a precise in-depth retrospective evaluation of the LRR pattern for patients treated with a TNT including an iHD-SBRT for localized PDAC, using for the first time a deformable image registration (DIR) technique.

## Materials and methods

### Patient selection

This study was approved by the Institutional Review Board of Institut Jules Bordet under the approval number CE3285.

As previously described [8], since August 2017, all patients with localized PDAC were prospectively treated according to a TNT strategy. The TNT included:

(1) Induction by modified FOLFIRINOX (mFFX: fluorouracil, irinotecan and oxaliplatin) or gemcitabine plus nab-Paclitaxel (Gem/nP; in case of intolerance or no response to mFFX after an intermediate restaging at 2 to 4 cycles) for ideally 6 cycles (a minimal number of 3 cycles was required);
(2) iHD-SBRT without concomitant chemotherapy
(3) Surgical exploration in case of no progression after a full restaging 4 to 7 weeks after the completion of iHD-SBRT.

If the localized tumor was deemed “never resectable” by the centralized multidisciplinary oncological board (MOC), iHD-SBRT was delivered as a definitive treatment and no further therapy was given until progression.

Eligible patients met the following criteria: biopsy-proven BR or LA adenocarcinoma; age ≥ 18 years, no evidence of metastatic disease at baseline or after induction chemotherapy; largest tumour diameter ⩽7cm; and normal renal, bone marrow and liver function.

BR and LA resection status were defined according to the NCCN criteria [17]. The resectability status had previously been assessed by a centralized (MOC) including dedicated pancreatic surgeons and radiologists.

### Stereotactic body radiation therapy protocol

The iHD-SBRT treatment was prepared and delivered as described in details in [8]. Briefly, fiducials markers were inserted in the tumour under endoscopic ultrasonography guidance minimum 5 days before the simulation [18]. Prior to the contrast CT scan, a 4 dimensional (4D)-CT scan was performed to assess respiratory motion. The use of an abdominal compression belt (ZiFix^TM^, QFix, Avondale, PA, USA) was required in case of fiducial respiratory motion > 5 mm in any direction, followed by a 4D-CT with the belt in place. A specialized radiologist systematically reviewed the contouring of the gross tumour volume (GTV). No clinical target volume (CTV) was delineated as elective nodes were not included in the treatment volume. Instead, we introduced the concept of a Tumour-Vessel Interface (TVI) volume. The TVI represents a focused, anatomically and biologically relevant region at high risk for microscopic disease spread, particularly along perivascular planes. As such, the TVI serves as a functional surrogate for a CTV in this context, consistent with ICRU definitions, but adapted to pancreatic SBRT. The TVI structure, internal target volume (ITV) and planned target volume (PTV) were created following the description in Table 1. An IDP was applied and therefore the dose prescription was not based on the target volume but based on OARs tolerance levels [19]. Tthe OARs dose constraints were applied as described in Table 2. The delivered dose was individually adapted and maximized to the highest achievable level in the PTV2 and particularly PTV3. iHD-SBRT was delivered using volumetric modulated arc therapy (VMAT) plans designed by the Monaco^TM^ planning system via Monte Carlo algorithm. Details about the surgery, adjuvant chemotherapy and follow-up were previously described in [8].

**Table 1:** Description of tumour-vessels interface, internal target volume and planned target volume.

Volume	Description
TVI	Whole circumference of major abdominal vessels in direct contact with the GTV
ITV_TVI_	TVI on all CT scan available, accounting for respiratory motion
ITV_GTV_	GTV on all CT scan available, accounting for respiratory motion
PTV1	ITV_TVI_ + ITV_GTV_ + 3 mm
PTV2	PTV1 – PRV of GI OAR
PTV3	ITV_TVI_ + 3 mm

TVI: tumour-vessels interface; GTV: gross tumour volume; ITV: internal tumour volume; PTV: planning target volume; PRV: planning organ at risk volume; GI OAR: Gastro-intestinal organs at risk, encounting duodenum, colon, small bowel and stomach.

**Table 2:** Main OARs dose constraints.

OAR	Dose constraint
GI_PRV (Duodenum, colon, small bowel, stomach)	D_max (0.5cc)_ < 35 Gy
	V_30Gy_ < 2cc

SpinalCord_PRV	V_20Gy_ < 1 cc
Kidney	D_mean_ < 10 Gy
	V_12Gy_ < 25 %

PRV: planning organ at risk volume; GI: Gastro-intestinal.

### Clinical outcomes

The primary outcome was to determined the LRR pattern after iHD-SBRT using DIR. Identification of LRR after iHD-SBRT was performed by experienced radiation oncologist and radiologists specialized in pancreatic cancer (MM, CB, JLE). Recurrences were identified on contrast-enhanced CT and/or MRI and/or positron emission tomography (PET-) CT. Any LRR progression meeting the RECIST criteria [20] or any recurrence after oncological surgery located between the diaphragm and L3 (excluding carcinomatosis and hepatic metastases) was considered as LRR.

### Deformable image registration

The initial RT planning contrast CT scan or MRI was considered as the primary imaging modality. The LRR after iHD-SBRT had to be clearly identified on the same imaging modality (planning [p]CT- recurrence [r]CT or dosimetry planning [p]MRI − recurrence [r]MRI) to be eligible for DIR analysis. The selected planning and recurrence images as well as the original structure sets were transferred trough the DICOM Collaboration system (DCMCollab, Odense University Hospital, Denmark) and imported into MIM software (MIM Software Inc. version 7.1.5, Cleveland, OH) for delineation, co-registration and uncertainty analysis.

The slice thickness of CT images was between 0.6 and 5 mm. For MRI, delineation was performed on T1 Fat-Sat or T2 haste weighted MRI sequences and the slide thickness was between 1.2 and 6.5 mm. (Supplementary Table 1).

All image datasets were imported into MIM in their native voxel resolution without interpolation. When needed, local registration refinements were manually performed using the “Reg Refine” tool to optimize alignment within the region of interest. For each case, regions of interest (ROIs) were systematically defined as the pancreatic segment(s) and the closest major abdominal vessel to the recurrence, based on anatomical proximity. This standardized approach ensured consistent anatomical reference points and did not systematically influence DIR quality metrics, as supported by DICE and MDA consistency across all cases.

Tumour recurrence was delineated on the corresponding (r)CT or (r)MRI (same sequence as [p]MRI). Regions of interest (ROIs) were delineated and included the closest pancreatic segment(s) and major abdominal arteries. For one case with a hepatic hilum recurrence, a liver ROI was also delineated. The segments of the pancreas (head, isthmus, body and tail) were delineated according to their anatomical definitions [21]. The major abdominal arteries were delineated as followed:

– Coeliac artery (CA): from its emergence from the aorta to its first bifurcation
– Superior mesenteric artery (SMA): from its emergence from the aorta till the first 2 cm
– Aorta: between the emergence of the celiac trunk and the emergence of renal arteries.

**Fig. 1:**
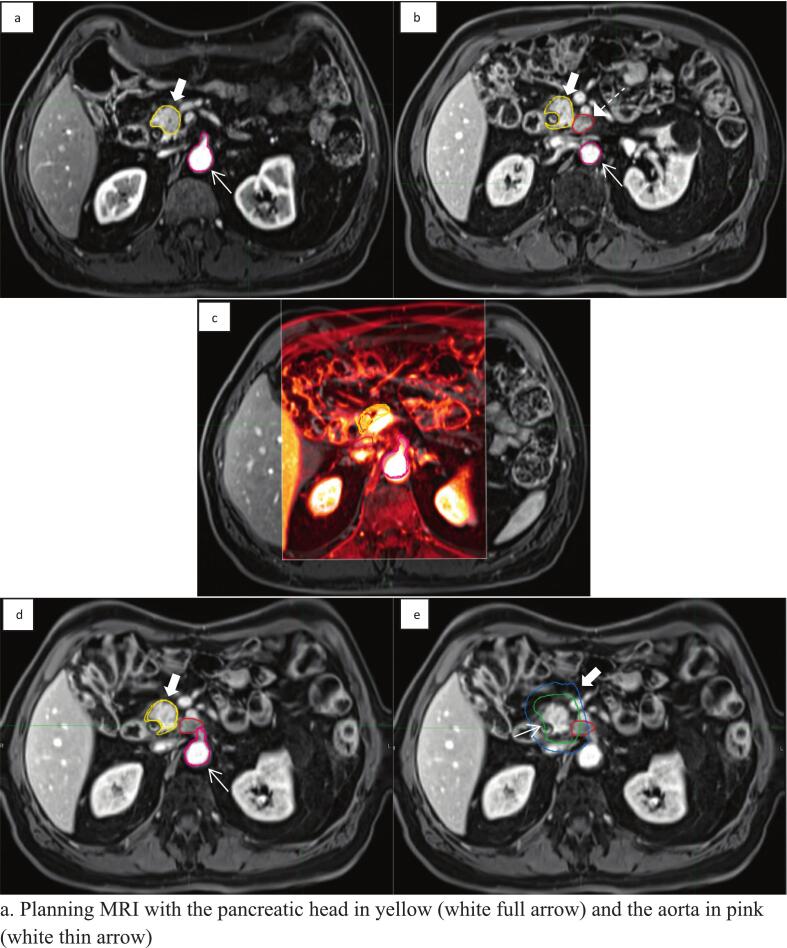
Deformable registration process. a. Planning MRI with the pancreatic head in yellow (white full arrow) and the aorta in pink (white thin arrow). b. Recurrence MRI with the pancreatic head in yellow (white full arrow), the aorta in pink (white thin arrow) and the loco-regional recurrence in red (white dashed arrow). c. Reg Refine Tool with a box centred on a Region of Interest, locking points of interest. d. Final deformed MRI, with both contours appearing on the image. e. Final deformed MRI, with the projected ID35 in green (thin arrow), the projected ID25 (full arrow) in blue and loco-regional recurrence in red. (For interpretation of the references to colour in this figure legend, the reader is referred to the web version of this article.)

The corresponding planning and recurrence imaging modalities were first matched using a rigid image registration (RIR) based on the closest ROIs from the recurrence (anatomical segments of the pancreas and/or a major abdominal artery). The MIM intensity-based algorithm was then performed, based on the previous rigid registration, focusing in the recurrence area [22]. In order to further improve quality, the MIM software “Reg Refine” tool was used to refine the local DIR manually and/or on local box-based rigid alignments. (Fig. 1) Briefly, this tool allows to visually assess the DIR and to correct the registration if needed by “locking” points of interest defined by the user as new initial conditions and a secondary DIR is then performed [22,23]. All the DIR were checked by a physician and physicist at the same time (MM, SP) and a specialized pancreatic radiation oncologist finally validated the DIR (CB).

### Evaluation of the DIR and the recurrence pattern

Dice similarity coefficient (DICE), Jacobian Index (JI), and Mean distance to agreement (MDA) were applied to analyse *per* patient uncertainties and similarities, including the DIR-propagated ROIs, and the quality of the DIR.

DICE allows to measure the spatial overlap and volume shared between two segmented anatomical structures after the registration process. The overlap of two structures is a good indicator of the registration accuracy between the two structures studied [24,25].

DICE was calculated following this formula:.$\mathrm{DICE}=2x\frac{volume\;of\;intersection\;fo\;A\;and\;B}{volume\;of\;A+volume\;of\;B}$

Interpretation of DICE results was performed following this scale: [26].

– Poor agreement: less than 0.2
– Fair agreement: 0.2 to 0.4
– Moderate agreement: 0.4 to 0.6
– Good agreement: 0.6 to 0.8
– Excellent agreement: 0.8 to 1.0

A Jacobian determinant (JD) was used to estimate the volume change during the DIR. A JD > 1 corresponds to a local tissue expansion in the ROI and a JD < 1 to a local tissue contraction [27,28].

MDA is calculated similarly to HD, except using mean distance across all points, and a high value reflects a large discrepancy [28].

The final visual result of each DIR was also reviewed by a pancreas expert physician (CB).

Finally, the 35 Gy isodose line (ID35), corresponding to a BED10 of 59.5 Gy, was projected on the DIR.

Recurrences were classified as:

• In-field (IF): > 50 % of recurrence volume within ID35
• Marginal (M): 20–50 % within ID35
• Out-of-field (OF): < 20 % or entirely outside ID35.

## Results

### Patient characteristics

Forty-one patients were treated by iHD-SBRT from January 2018 to January 2021. The baseline and clinical characteristics of the whole cohort were previously described in [29].

Briefly, the rates of BR and LA tumours were respectively 46.3 (n = 19) and 53.7 % (n = 22). The median number of chemotherapy cycles was 7 (IQR 6 – 8) and the median duration of induction chemotherapy was 3.7 months (IQR 2.6 – 4.6). After TNT, an oncological resection was performed in 46.3 % (n = 19).

Among these 41 patients, a LLR was identified in 18 patients (43.9 %). LRR alone was the first type of recurrence in 14.6 % (n = 6/41) of the cases whereas LRR and distant metastasis occurred simultaneously in 12.2 % (n = 5/41). For 17.1 % of the patients (n = 7/41), LLR occurred after the occurrence of distant metastasis (Supplementary Fig. 1). The baseline and clinical characteristics of the LRR cohort is described in Supplementary Table 2. For 17 of these patients, corresponding imaging modalities ([p]CT-[r]CT or [p]MRI-[r]MRI) were available and suitable for deformable image registration (DIR) analysis. One patient was excluded due to incompatible imaging modalities. These patients were prospectively treated but are retrospectively analyzed in this study. Previously published outcome data on 39 patients [8] overlap partially with the cohort included here.

### Evaluation of loco-regional recurrence in the iHD-SBRT cohort

**Fig. 2:**
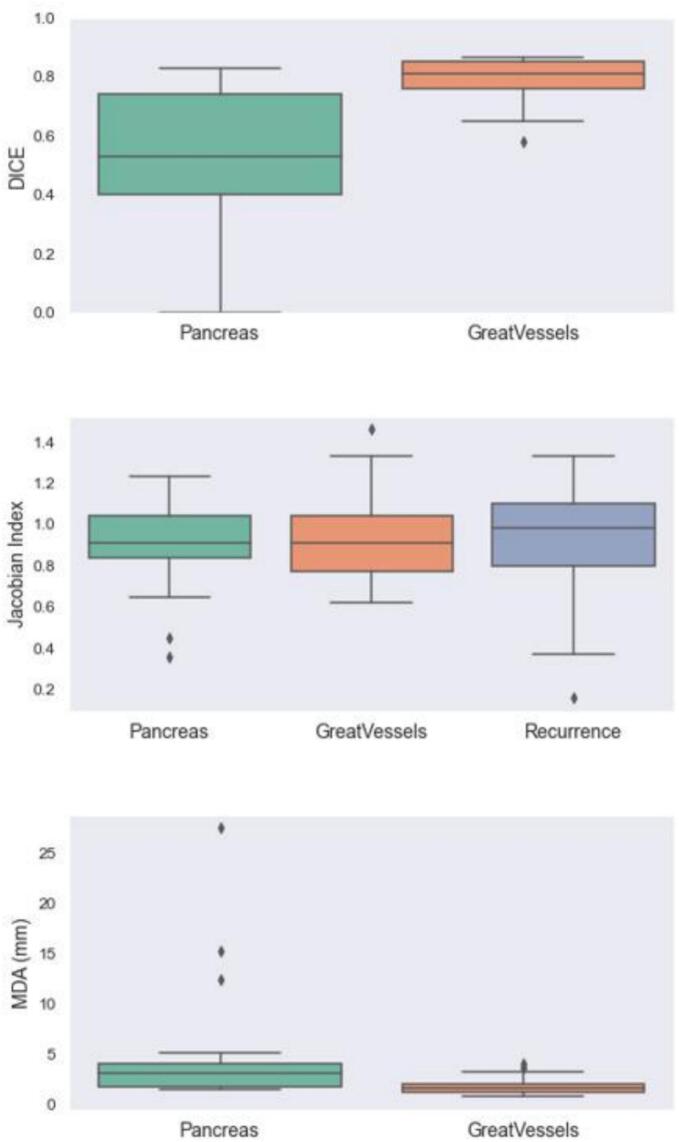
Dice similarity coefficient, Jacobian Index and Mean distance to agreement (MDA).

Adequate corresponding imaging modalities ([p]CT- [r]CT or [p]MRI − [r]MRI) were available for DIR analyses for 17 cases. The values obtained from each ROIs for the three metrics evaluated (DICE, MDA and JD) are described in details in Table 3 and in Fig. 2. Before calculating the different metrics, each DIR was reviewed by two radiation oncologists (MM, CB) to see if the result was logical and clinically relevant, and then validated. From the ID35, recurrences were identified as OF in 53 % (n = 9/17), M in 23.5 % (n = 4) and IF in 23.5 % (n = 4) of the cases. (Supplementary Fig. 2) The detailed anatomical localisation of all the LRR is described in the Table 4.

**Table 3:** Results of the four metrics used to analyze the DIR (n = 17).

	**Metrics (mean value +/- SD)**			
**Structures − ROI**	**DICE**	**MDA (mm)**	**JD**	**Visualization**
*Pancreas*	0.49 +/- 0.26	5.52 +/- 6.84	0.89 +/- 0.23	Pass
*Abdominal arteries*	0.78 +/- 0.08	1.87 +/- 0.95	0.94 +/- 0.20	Pass
*Recurrence*	/	/	0.89 +/- 0.19	Pass

DIR = deformable image registration, SD = standard deviation; ROI = region of interest; DICE = Dice similarity coefficient; HD= Hausdorff distance; MDA = Mean distance to agreement; JD = Jacobian Index.

**Table 4:** Anatomical location of the loco-regional recurrences (n = 17).

ID35 (BED_10_: 59.5 Gy)	Location	N
OF	Remaining pancreas (post-surgery)	3
	Hepatic hilum (or within 1 cm)	3
	Para-aortic adenopathy	3

M	Close to SMA (in contact or within 5 mm) Close to CA (in contact or within 5 mm) Hepatic hilum	11 2

IF	Primitive PDAC progression	4

ID35 = isodose line 35 Gy; BED_10_: biological equivalent dose (α/β 10); OF = outside-the-field; IF = in field; SMA = superior mesenteric artery; PDAC = pancreatic ductal adenocarcinoma; CA = coeliac artery.

## Discussion

The impact of locoregional recurrence (LRR) on the morbidity and mortality of pancreatic ductal adenocarcinoma (PDAC) is considerable. Approximately one-third of patients die from locoregional progression rather than distant metastases [30]. Surgery with R0 resection remain essential for curative intent. Itnot only improve overall survival but also enhance local control [31]. Historically, the rate of local recurrence after surgery was high (50–90 %). Withadjuvant treatments, this rate has reduced this rate to about 50 % [32].

To increase the R0 resection rate, several neoadjuvant treatment protocols – combining chemotherapy and radiochemotherapy − have been evaluated. Phase II studies have reported promising results. However, a phase III trial comparing surgery followed by adjuvant chemotherapy versus the addition of neoadjuvant chemoradiotherapy showed only a modest survival gain (median difference of 1.5 months). A *meta*-analysis based on intention-to-treat data confirmed a survival benefit for neoadjuvant treatment, though it did not distinguish between chemotherapy and chemoradiotherapy [33,34]. Overall, evidence for an improvement in overall survival remains limited. Nevertheless, ESMO guidelines recommend induction therapy for borderline and locally advanced tumors to improve R0 resection and local control, although the optimal induction strategy is still under debate [35,36].

After neoadjuvant SBRT with high BED, reported LRR rates range from 40 % to 60 %, consistent with our findings (43 %) [12,37]. In terms of local control, High BED SBRT demonstrated superior outcomes compared to radiochemotherapy (1-year LC: 75.8 % [SBRT] vs 39.3 % [RCT], p = 0.004).

Detailed clinical outcomes for the entire cohort, including R0 resection rate, local control, and overall survival, have been previously reported in our previous study (Manderlier et al., *Cancers*, 2022) [29]. The present analysis focuses specifically on the loco-regional recurrence patterns within that population

For 17 patients, the LRR was clearly identified on the same imaging modality, allowing a DIR analysis using three metrics: DICE, MDA and JD. Two ROIs were delineated for each case: 1/ the pancreas segment closest to the LRR; 2/ the nearest major abdominal artery, as vascular structures are known to be reliable landmarks for pancreatic registration [38]. Depending on the patient, the LRR could be adjacent to the pancreas and/or major vessels.

For each patient, both rigid image registration (RIR) and DIR were independently evaluated to determine the most clinically relevant alignment. The two ROIs were not always used simultaneously for guidance. Across all patients, registration based on the great vessels showed good agreement, with mean DICE = 0.78 ± 0.08 and mean MDA < 2 mm – within AAPM TG-132 tolerances [39]. These findings confirm that major abdominal vessels are suitable landmarks for pancreatic DIR.

For the pancreatic ROI, the results showed moderate agreement (mean DICE = 0.49 ± 0.26; mean MDA ≈ 5 mm). When registration was guided by the vessels rather than the pancreas, DICE values decreased and MDA increased for the pancreas structure. Conversely, when the pancreas served as the primary ROI, registration quality improved. The lower accuracy for pancreatic registration likely reflects its greater mobility (e.g., respiratory motion, abdominal compression during imaging) and deformation over time (e.g., parenchymal atrophy, inflammation, or ductal dilation due to obstruction).

JD analysis indicated a shrinkage tendency for both the pancreas (0.89 ± 0.23) and the LRR (0.89 ± 0.19) and an expected mean value closer to 1 for the vessels (0.94 +/- 0.20). The shrinkage is expected: LRR volumes were absent on initial [p]CT or [p]MRI scans, and the pancreas often decreases in size after treatment. Possible explanations include reduced tumor-related inflammation, relief of ductal obstruction (especially post-surgery), and radiation-induced volume loss [40].

Based on the DIR results, the ID35 was projected and LRRs were categorized as OF, M and IF in 53 %, 23.5 % and 23.5 % of cases, respectively. All in-field recurrences were located within well-covered high-dose regions (≥ 35 Gy isodose, BED10 ≈ 60 Gy), excluding underdosage as a cause. These failures thus likely reflect intrinsic radioresistance of PDAC, possibly driven by hypoxia, dense desmoplastic stroma, and KRAS/TGFβ pathway activation, as previously reported [[41], [42], [43], [44]]. Among the remaining 13 cases, two out-of-field (OF) recurrences occurred at the hepatic hilum – outside the irradiated field due to the absence of elective nodal coverage – representing < 5 % of the total cohort (2/41). Both cases corresponded to tumors located in the pancreatic head, for which the hepatic hilum constitutes a known lymphatic drainage pathway. These OF events most likely represent regional lymphatic spread beyond the treated volume rather than true radioresistance. Indeed, the hepatic hilum was not included in the tumor-vessel interface (TVI) structure or in any elective nodal coverage, supporting the hypothesis of a geographical miss rather than failure of dose escalation.The evidence supporting elective nodal irradiation in PDAC is weak outside the adjuvant setting. Most SBRT studies suggest that delivering a higher BED_10_ offers greater benefit than expanding the treated volume [7,45]. The six additional OF recurrences were located either at the surgical margin or in *para*-aortic nodes.

Marginal recurrences were located within 5 mm of the SMA or CA, confirming these as high-risk areas after R1 resection or SBRT [31,46]. The four marginal cases were carefully reviewed to assess potential improvements in target volume delineation. Previous SBRT studies have raised similar concerns about undercoverage of high-risk perivascular regions [12,47,48] (Supplementary Table 3). To mitigate the risk, we propose expanding the target the target volume to include the full involved vessel circumference plus a 5-mm radial margin. However, this recommendation is hypothesis-generating and should not be interpreted as a validated DIR-based delineation change. Prospective evaluation is ongoing in the STEREOPAC trial [15].

To our knowledge, this is the first study employing DIR to map LRR patterns in pancreatic cancer. Typically, LRR mapping is performed on a reference CT of a healthy subject, which introduces errors due to anatomical variability [12,47]. Another approach involves rigid fusion between the dosimetric CT and the first recurrence CT [43]. DIR offers a major advantage by accounting for patient-specific anatomical changes, especially after surgery or significant weight loss. It enables a more precise estimation of recurrence distance from critical structures, such as vessels. DIR has already proven valuable in validating the European Society for Radiotherapy and Oncology (ESTRO) consensus guidelines for breast cancer. [49].

In conclusion, due to the presence of four marginal recurrences around major abdominal vessels, our internal iHD-SBRT protocol has been adapted to ensure a better coverage of the vessels close to the primary tumour in order to reduce the risk of marginal recurrences without causing a major increase of the treated volume. The randomized phase II STEREOPAC trial [NCT05083247] is ongoing, aiming to compare the best neoadjuvant therapeutic option between mFFX alone versus mFFX + iHD-SBRT in 256 BR patients and theLRR pattern after iHD-SBRT will be further studied as a secondary endpoint [15].

## Disclosures

The authors have no conflicts of interest to declare.

## Author contributions

All authors reviewed and edited the manuscript, gave critical input and gave final approval for publication.

MM delineated the recurrence and ROI of all patients, performed RIR and DIR and evaluated the localisation of the recurrence compared to the ID35.

SP performed RIR and DIR evaluated the DIR using DICE, JI and MDA.

JLE helped to identify the local recurrence if the case was complicated or unclear.

AG helped to implement and develop the DIR.

CB helped to delineated the local recurrence and validated the final version of the DIR.

## CRediT authorship contribution statement

**Martin Manderlier:** Conceptualization, Methodology, Validation, Formal analysis, Investigation, Data curation, Writing – original draft. **Sara Poeta:** Conceptualization, Validation, Formal analysis, Writing – review & editing, Visualization. **Jean-Luc Engelholm:** Validation, Writing – review & editing. **Akos Gulyban:** Software, Writing – review & editing. **Jean-Luc Van Laethem:** Resources, Supervision, Writing – review & editing. **Christelle Bouchart:** Conceptualization, Investigation, Validation, Formal analysis, Supervision, Writing – review & editing.

## Funding

*The last author disclosed receipt of the following financial support for the research:* This work was supported by a doctoral grant from the “Amis de l’Institut Bordet” [grant number: 2021–03] and by the “Fonds de la Recherche Scientifique – FNRS” [Grant number FC 33593] (CB).

## Declaration of Competing Interest

The authors declare that they have no known competing financial interests or personal relationships that could have appeared to influence the work reported in this paper.

## References

[1] Kleeff J., Korc M., Apte M. (2016). Pancreatic cancer. Nat Rev Dis Primers.

[2] Ferlay J. (2021). Cancer statistics for the year 2020: an overview. Int J Cancer.

[3] Balaban E.P., Mangu P.B., Khorana A.A. (2016). Locally advanced, unresectable pancreatic cancer: American society of clinical oncology clinical practice guideline. J Clin Oncol.

[4] Konstantinidis IT, Warshaw AL, Allen et al. Pancreatic Ductal Adenocarcinoma: Is There a Survival Difference for R1 Resections Versus Locally Advanced Unresectable Tumors? What Is a “True” R0 Resection? Annals of Surgery. avr 2013;257(4):731–6. 10.1097/SLA.0b013e318263da2f 22968073

[5] Gnerlich JL, Luka SR, Deshpande AD et al. Microscopic Margins and Patterns of Treatment Failure in Resected Pancreatic Adenocarcinoma. Arch Surg. 1 août 2012;147(8):753. 10.1001/archsurg.2012.1126 22911074

[6] Delpero J.R., Bachellier P., Regenet N., Le Treut Y.P., Paye F., Carrere N. (2014). Pancreaticoduodenectomy for pancreatic ductal adenocarcinoma: a French multicentre prospective evaluation of resection margins in 150 evaluable specimens. HPB.

[7] Bouchart C., Navez J., Closset J. (2020). Novel strategies using modern radiotherapy to improve pancreatic cancer outcomes: toward a new standard?. Ther Adv Med Oncol.

[8] Bouchart C., Engelholm J.L., Closset J. (2021). Isotoxic high-dose stereotactic body radiotherapy integrated in a total multimodal neoadjuvant strategy for the treatment of localized pancreatic ductal adenocarcinoma. Ther Adv Med Oncol.

[9] Katz M.H., Shi Q., Meyers J., Herman J.M., Chuong M., Wolpin B.M. (2022). Efficacy of preoperative mFOLFIRINOX vs. mFOLFIRINOX plus hypofractionated radiotherapy for borderline resectable adenocarcinoma of the pancreas: the A021501 phase 2 randomized clinical trial. JAMA. Oncol.

[10] Zhu X., Shi D., Li F. (2018). Prospective analysis of different combined regimens of stereotactic body radiation therapy and chemotherapy for locally advanced pancreatic cancer. Cancer Med.

[11] Rudra S., Jiang N., Rosenberg S.A. (2019). Using adaptive magnetic resonance image-guided radiation therapy for treatment of inoperable pancreatic cancer. Cancer Med.

[12] Zhu X., Cao Y., Su T. (2020). Failure patterns and outcomes of dose escalation of stereotactic body radiotherapy for locally advanced pancreatic cancer: a multicenter cohort study. Ther Adv Med Oncol.

[13] Arcelli A., Guido A., Buwenge M. (2020). Higher biologically effective dose predicts survival in SBRT of pancreatic cancer: a multicentric analysis (PAULA-1). Anti Can Res.

[14] Saúde-Conde R., El Ghali B., Navez J., Bouchart C., Van Laethem J.L. (2024). Total neoadjuvant therapy in localized pancreatic cancer: is more better?. Cancers (Basel).

[15] Bouchart C., Navez J., Borbath I., Geboes K., Vandamme T., Closset J. (2023). Preoperative treatment with mFOLFIRINOX or Gemcitabine/Nab-paclitaxel +/- isotoxic high-dose stereotactic body Radiation Therapy (iHD-SBRT) for borderline resectable pancreatic adenocarcinoma (the STEREOPAC trial): study protocol for a randomised comparative multicenter phase II trial. BMC Cancer.

[16] Kharofa J., Mierzwa M., Olowokure O. (2019). Pattern of marginal local failure in a phase II trial of neoadjuvant chemotherapy and stereotactic body radiation therapy for resectable and borderline resectable pancreas cancer. Am J Clin Oncol.

[17] Tempero M.A., Malafa M.P., Al-Hawary M. (2021). Pancreatic adenocarcinoma, version 2.2021, NCCN clinical practice guidelines in oncology. J Natl Compr Canc Netw.

[18] Figueiredo M., Bouchart C., Moretti L. (2021). EUS-guided placement of fiducial markers for stereotactic body radiation therapy in pancreatic cancer: feasibility, security and a new quality score. EIO.

[19] Zindler J.D., Thomas C.R., Hahn S.M. (2015). Increasing the therapeutic ratio of stereotactic ablative radiotherapy by individualized isotoxic dose prescription. J Natl Cancer Inst.

[20] Eisenhauer E.A., Therasse P., Bogaerts J. (2009). New response evaluation criteria in solid tumours: revised RECIST guidelines (version 1.1). Eur J Cancer.

[21] Casillas J., Levi J.U., Quiroz A. (2016).

[22] Calusi S., Labanca G., Zani M. (2019). A multiparametric method to assess the MIM deformable image registration algorithm. J Appl Clin Med Phys.

[23] Johnson P.B., Padgett K.R., Chen K.L., Dogan N. (2016). Evaluation of the tool “Reg Refine” for user-guided deformable image registration. J Appl Clin Med Phys.

[24] Dice L.R. (1945). Measures of the amount of ecologic association between species. Ecology.

[25] Woerner A.J., Choi M., Harkenrider M.M. (2017). Evaluation of deformable image registration-based contour propagation from planning CT to Cone-Beam CT. Technol Cancer Res Treat.

[26] Akbarzadeh A., Gutierrez D., Baskin A., Ay M.R., Ahmadian A., Riahi Alam N. (2013). Evaluation of whole-body MT to CT deformable image registration. J Appl Clin Med Phys.

[27] Jurkovic I.A., Papanikolaou N., Stathakis S. (2020). Objective assessment of the quality and accuracy of deformable image registration. Journal of Medical Physics.

[28] Brock KK, Mutic S, McNutt et al. Use of image registration and fusion algorithms and techniques in radiotherapy: report of the AAPM radiation therapy committee Task Group No. 132. Med Phys. 2017; 44:e43-e76. 10.1002/mp.12256 28376237

[29] Manderlier M., Navez J., Hein M., Engelholm J.L., Closset J., Bali M.A. (2022). Isotoxic high-dose stereotactic body radiotherapy (iHD-SBRT) versus conventional chemoradiotherapy for localized pancreatic cancer: a single cancer center evaluation. Cancers (Basel).

[30] Iacobuzio-Donahue CA, Fu B, Yachida S et al. DPC4 Gene Status of the Primary Carcinoma Correlates With Patterns of Failure in Patients With Pancreatic Cancer. JCO. 10 avr 2009;27(11):1806–13. 10.1200/JCO.2008.17.7188 PMC2668706 19273710

[31] Kalisvaart M., Broadhurst D., Marcon F. (2020). Recurrence patterns of pancreatic cancer after pancreatoduodenectomy: systematic review and a single-centre retrospective study. HPB.

[32] Boyle J., Czito B., Willett C., Palta M. (2015). Adjuvant radiation therapy for pancreatic cancer: a review of the old and the new. J Gastrointest Oncol.

[33] Versteijne E., van Dam J.L., Suker M. (2022). Neoadjuvant chemoradiotherapy versus upfront surgery for resectable and borderline resectable pancreatic cancer: long-term results of the dutch randomized PREOPANC trial. J Clin Oncol.

[34] Katz, Matthew H. G., Qian Shi et al. Preoperative Modified FOLFIRINOX Treatment Followed by Capecitabine-Based Chemoradiation for Borderline Resectable Pancreatic Cancer: Alliance for Clinical Trials in Oncology Trial A021101. JAMA Surgery 151, no. 8 (17 August 2016): e161137. 10.1001/jamasurg.2016.1137 PMC5210022 27275632

[35] Conroy, T. et al. Pancreatic cancer: ESMO Clinical Practice Guideline for diagnosis, treatment and follow-up. Annals of Oncology, Volume 34, Issue 11, 987 – 1002. 10.1016/j.annonc.2023.08.009 37678671

[36] Saúde-Conde R., El Ghali B., Navez J., Bouchart C., Van Laethem J.-L. (2024). Total neoadjuvant therapy in localized pancreatic cancer: is more better?. Cancers.

[37] Shin Y.S., Park H.H., Park J.H. (2022). Stereotactic body radiation therapy versus concurrent chemoradiotherapy for locally advanced pancreatic cancer: a propensity score-matched analysis. Cancers (Basel).

[38] Karasawa K., Oda M., Kitasaka T. (2017). Multi-atlas pancreas segmentation: atlas selection based on vessel structure. Med Image Anal.

[39] Brock K.K., Mutic S., McNutt T.R., Li H., Kessler M.L. (2017). Use of image registration and fusion algorithms and techniques in radiotherapy: Report of the AAPM Radiation Therapy Committee Task Group No. 132. Med Phys.

[40] Gemici C., Yaprak G., Ozdemir S., Baysal T., Seseogullari O.O., Ozyurt H. (2018 Dec 3). Volumetric decrease of pancreas after abdominal irradiation, it is time to consider pancreas as an organ at risk for radiotherapy planning. Radiat Oncol.

[41] Seshacharyulu P., Baine M.J., Souchek J.J. (2017). Biological determinants of radioresistance and their remediation in pancreatic cancer. BBA.

[42] Hu B., Ma X., Huang R. (2021). Identification of key genes mutations associated with the radiosensitivity by whole exome sequencing in pancreatic cancer. Front Oncol.

[43] Baine M.J., Sleightholm R., Lin C. (2019). Incidence and patterns of locoregional failure after stereotactic body radiation therapy for pancreatic adenocarcinoma. Practical Radiation Oncology Janv.

[44] Benkhaled S., Peters C., Jullian N., Arsenijevic T., Navez J., Van Gestel D. (2023). Combination, modulation and interplay of modern radiotherapy with the tumor microenvironment and targeted therapies in pancreatic cancer: which candidates to boost radiotherapy?. Cancers (Basel).

[45] Brunner T.B., Haustermans K., Huguet F. (2021). ESTRO ACROP guidelines for target volume definition in pancreatic cancer. Radiother Oncol.

[46] Dholakia A.S., Kumar R., Raman S.P. (2013). Mapping patterns of local recurrence after pancreaticoduodenectomy for pancreatic adenocarcinoma: a new approach to adjuvant radiation field design. Int J Radiat Oncol Biol Phys.

[47] Zhu X., Ju X., Cao Y. (2019). Patterns of local failure after stereotactic body radiation therapy and sequential chemotherapy as initial treatment for pancreatic cancer: implications of target volume design. Int J Radiat Oncol Biol Phys.

[48] Barrord M., Ahmad S., Patel S. (2020). Patterns of failure after neoadjuvant stereotactic body radiation therapy or fractionated chemoradiation in resectable and borderline resectable pancreatic cancer. Pancreas.

[49] Chang J.S., Lee J., Chun M., Shin K.H., Park W., Lee J.H. (2018). Mapping patterns of locoregional recurrence following contemporary treatment with radiation therapy for breast cancer: a multi-institutional validation study of the ESTRO consensus guideline on clinical target volume. Radiotherapy and Oncology Janv.

