# Supplementary material for: Evaluation of loco-regional recurrences using deformable image registration after isotoxic high dose stereotactic body radiotherapy in localised pancreatic cancer
Source: Clin Transl Radiat Oncol. 2025 Nov 19;56:101081. doi: 10.1016/j.ctro.2025.101081 (PMC12720030; doi:10.1016/j.ctro.2025.101081)
Supplement: Supplementary Data 1 [file mmc1.docx]

**SUPPLEMENTARY TABLE AND FIGURES**

**Supplementary Table 1** - Details of the radiological images acquisitions for the primary and recurrent PDAC.

| **Patient** | **CT** | | **MRI** | | | |
| --- | --- | --- | --- | --- | --- | --- |
|  | **Primary** | **Recurrence** | **Primary** | | **Recurrence** | |
|  | Slide thickness (mm) | Slide thickness (mm) | Slide thickness (mm) | Sequence | Slide thickness (mm) | Sequence |
| 1 | 1 | 5 | / | / | / | / |
| 2 | 3 | 1.5 | / | / | / | / |
| 3 | 1 | 5 | / | / | / | / |
| 4 | 2 | 2 | / | / | / | / |
| 5 | 1 | 0.7 | / | / | / | / |
| 6 | 1.6 | 5 | / | / | / | / |
| 7 | 1.6 | 1.25 | / | / | / | / |
| 8 | 2 | 0.6 | / | / | / | / |
| 9 | 2 | 0.8 | / | / | / | / |
| 10 | 1.6 | 1 | / | / | / | / |
| 11 | / | / | 1.75 | T1 Fat-Sat | 1.75 | T1 Fat-Sat |
| 12 | / | / | 1.2 | T1 Fat-Sat | 1.4 | T1 Fat-Sat |
| 13 | / | / | 1.2 | T1 Fat-Sat | 1.2 | T1 Fat-Sat |
| 14 | / | / | 1.2 | T1 Fat-Sat | 1.2 | T1 Fat-Sat |
| 15 | / | / | 5 | T2 HASTE | 5.5 | T2 HASTE |
| 16 | / | / | 5 | T2 HASTE | 5.5 | T2 HASTE |
| 17 | / | / | 6.5 | T2 HASTE | 5.5 | T2 HASTE |

CT= computed tomography; MRI: magnetic resonance imaging; Fat-sat: fat saturation; T1: T1 weighted image; T2: T1 weighted image; HASTE: half Fourier single shot turbo spin echo

**Supplementary Table 2** - Baseline and clinical characteristics of the patients with LRR.-

|  | Recurrence group (n=18) |
| --- | --- |
| ***Gender***  Female (n=5)  Male (n=13) | 27.8%  72.2% |
| ***Age (years)***  <60 (n=9)  ≥60 (n=9) | 50.0%  50.0% |
| ***CA19.9 values at diagnosis (kU/L)***  <200 (n=10)  ≥200 (n=8) | 55.6%  44.4% |
| ***Primary Site***  Head/uncus/isthmus (n=15)  Body/tail (n=3) | 83.3%  16.7% |
| ***Tumour diameter (mm)***  <40 (n=9)  ≥40 (n=9) | 50.0%  50.0% |
| ***Staging TNM 8^th^ ed.***  IB (n=1)  II A/B (n=4)  III (n=13) | 5.6%  22.2%  72.2% |
| ***Resection status***  Borderline (n=7)  Locally advanced (n=11) | 38.9%  61.1% |
| ***Number of chemotherapy cycles (induction)***  0-3 (n=0)  4-8 (n=14)  >8 (n=4) | 0.0%  77.8%  22.8% |
| ***Time of induction (months)***  <2 (n=0)  ≥2 - <4 (n=10)  ≥4 (n=8) | 0.0%  55.6%  44.4% |
| ***Oncological resection***  No (n=8)  Yes (n=10) | 44.4%  55.6% |
|  |  |
| *Age (years), median [IQR]* | 61.4 (51.9 – 70.0) |
| *CA19.9 value at diagnosis (kU/L), median [IQR]* | 82.0 (14.3 – 211.0) |
| *Tumour diameter (mm), median [IQR]* | 37.5 (32.3 – 45.8) |
| *Number of chemotherapy cycles (induction),* *median [IQR]* | 7.5 (6 – 8) |
| *Time of induction (months), median [IQR]* | *3.7 (3.3 – 4.5)* |

**Supplementary Table 3** - Comparison of our pattern of failure with modern pancreatic SBRT trials available in the literature.

| **Study** | **Study design** | **Type of RT** | **N** | **N of local failure** | **Dose (Gy/#)** | **Median BED_10_ (Gy)** | **Chemotherapy** | **Local failure alone (%)** | **Mapping of recurrence (n)** | **Local failure and distant metastasis (%)** | **Mapping of recurrence (n)** | **Median OS (months)** | **Median PFS (months)** |
| --- | --- | --- | --- | --- | --- | --- | --- | --- | --- | --- | --- | --- | --- |
| Current study | Retrospective | SBRT | 41 | 18 | 35-40/5 (SIB TVI up to 53Gy) | 73.8 | I: Gem-NP or mFFX | 14.6 | IF: 2*  M: 1  OF: 3 | 29.3 | IF: 2  M: 3  OF: 6 | 24.7 | 14.6 |
| Baine et al. 2018^43^ | Retrospective | SBRT | 69 | 22 | 35/5 | 59.5 | I: ND  C: 5FU, Capecitabine, Nelfinavir or none | 31.9 | IF: 11  IOF: 7  OF: 4 | ND | ND | 15 | 9 |
| Zhu et al. 2019^48^ | Retrospective | SBRT | 510 | 510 | 37/5-8 | 64.35 | Post-R: Gem or S-1 for 4-6 cycles | 42.5 | IF: 117^§^  OF: 100 | 57.5 | IF: 51  IOF: 67  MOF: 112  OF: 63 | ND | ND |
| Kharofa et al. 2019^16^ | Phase 2 | SBRT | 18 | 9 | 33/5 | 54.78 | I: Gem-NP or FFX for 3 months | 27.8 | ND | 22.2 | ND | 21 | 11 |
| Zhu et al. 2020^12^ | Retrospective | SBRT | 972 | Arm A: 486 | 37/5-8 | 64.38 | Post-RT: Gem or S-1 for 4-6 cycles | 63.6 | IF: 97^§^  M: 109  OF: 103 | 36.4 | IF: 86  OF: 91 | 18.2 | 13.3 |
|  |  |  |  | Arm B: 486 | 42/5-8 | 74.62 |  | 57.6 | IF: 72^§^  M: 84  OF: 124 | 42.4 | IF: 101  OF: 105 | 20.3 | 15.4 |
| Barrord et Al. 2020^49^ | Retrospective | SBRT | 18 | 6 | 33/5 | 54.78 | I: Gem-NP or FFX | 22.2^#^ | ND | 11.1 | ND | 19.3 | 11 |
| Libbey et al. 2024^51^ | Retrospective | SBRT | 22 | 5 | 30/5 (SIB up to 45Gy) | ND | I: FFX, FOLFOX, Gem-NP or other | 0 | / | 5 | IF: 2 ^α^  M: 2  MIF: 1 | ND | ND |

BED : Biologically effective dose; OS : overall survisal; PFS : progression-free survival; SBRT : stereotactic body radiotherapy; I : induction; Gem-NP : gemcitabine/nab-paclitaxel; mFFX : modified FOLFIRINOX; IF : in-field recurrence; M : marginal recurrence; OF : out-field recurrence; post-RT : post-radiotherapy; Gem : gemcitabine; S-1 : oral 5-fluorouracil prodrug; FFX : FOLFIRINOX; ND : not described in the study; CRT : chemo-radiotherapy; IOF : in and out-field recurrence; MOF : marginal and out-field recurrence; C : concurrent chemotherapy; MIF : marginal and in-field recurrence

*. IF : > 50% of the LR in the ID35; M : 20-50% of the LR in the ID35; OF : < 20% of the LR in the ID35

§. IF : > 80% of the LR in the prescription dose line; M : 20-80% of the LR in the prescription dose line; OF : < 20% of the LR in the prescription dose line

#. first site of failure only described

α. IF : within the prescription isodose line ; M : within 50% of the prescription isodose line

**Supplementary figure 1** - Timing of locoregional recurrence following the surgical status.

**Supplementary figure 2** - Localisation of locoregional recurrence following the surgical status.

**Supplementary figure 3** - Update of contouring guidelines**
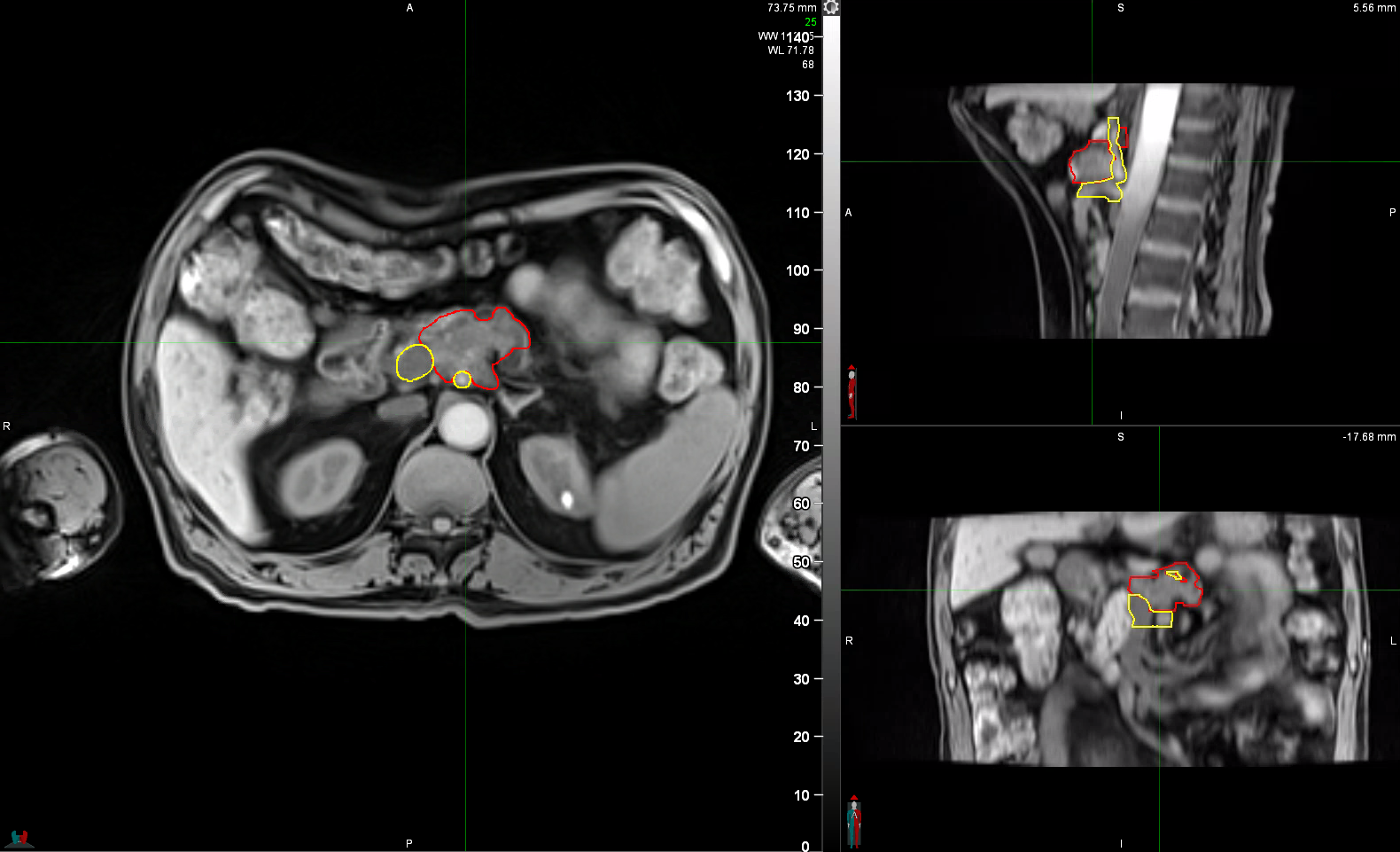
**

GTV in red, TVI in yellow.
